# Draft genome sequence of *Marinobacter* sp. DUT-3, a manganese-oxidizing and potential antibiotic-resistant bacterium from Bohai coastal sediments

**DOI:** 10.1128/mra.00511-25

**Published:** 2025-10-13

**Authors:** Tongtong Wu, Jieyi Li, Hao Zhou, Hongzhi Tang, Haixia Pan

**Affiliations:** 1 Key Laboratory of Industrial Ecology and Environmental Engineering (Ministry of Education), School of Chemical Engineering, Ocean and Life Sciences, Panjin Campus, Dalian University of Technology 506955, Panjin, China; 2 Guangxi Key Laboratory of Beibu Gulf Marine Resources, Environment and Sustainable Development, Fourth Institute of Oceanography, Ministry of Natural Resources 635955, Beihai, China; University of Southern California, Los Angeles, California, USA

**Keywords:** manganese-oxidizing bacteria, genome sequencing, antibiotic resistance genes, marine sediments, *Marinobacter*

## Abstract

A manganese-oxidizing bacterium, *Marinobacter* sp. DUT-3, was isolated from Bohai coastal sediments. A total of 24 contigs with GC content of 57.91% and 3,817 protein-coding genes were obtained by genome sequencing. Isolation of this strain suggests potential for synergistic antibiotics removal via biogenic manganese oxides and intrinsic resistance.

## ANNOUNCEMENT

Manganese-oxidizing bacteria (MnOB) have been reported to directly oxidize Mn(II) to Mn(III)/Mn(IV) through various enzymes, such as multicopper oxidases and animal heme peroxidases (1). The biogenic manganese oxides generated by bacteria were considered as both oxidants and absorbents and also reported to degrade organic pollutants such as the antibiotic ciprofloxacin and polycyclic aromatic hydrocarbons through oxidation or adsorption (2–4).

In this study, a manganese-oxidizing bacterial strain, DUT-3, was isolated from coastal surface sediments of the Bohai Bay (40.83°N, 121.30°E) (China). One gram of sediment was homogenized in 9 mL sterile artificial seawater (35 g/L NaCl) by vortexing for 10 min, serially diluted 2× and 100× with the same artificial seawater, then 100 µL of each dilution was spread on solid modified Haloarchaeal medium (20 g/L agar added) and incubated at 28°C for 7 days, and then brown colonies were picked and further inoculated into liquid modified Haloarchaeal medium (5). The content of medium is as follows (per liter): 0.05 g yeast extract, 0.25 g peptone, 1.0 g sodium pyruvate, 5.4 g KCl, 0.429 g NaNO_3_, 13.4 g MgSO_4_·7H_2_O, 11.5 g MgCl_2_·6H_2_O, 35.0 g NaCl, 0.3 g K_2_HPO_4_, 0.29 g CaCl_2_, and 100 µM MnCl_2_ (pH 7.0–7.2). The pure colonies changed to blue after reacting with leucoberbelin blue solution and were selected as MnOB (3). The DNA of strain DUT-3 was extracted with Ezup Column Bacteria Genomics DNA Purification Kit (Sangon, Shanghai, China). The universal primers 27F (5′-AGAGTTTGATCCTGGCTCAG) and 1492R (3′-TACGGCTACCTTGTTACGACTT) were used for PCR amplification (6). The obtained 16S rRNA sequences were identified in the EzBiocloud database (https://www.ezbiocloud.net/) (7). The results show that strain DUT-3 shares 98.91% identity to *Marinobacter lipolyticus* SM19 (GenBank no. ASAD01000031), suggesting that strain DUT-3 is affiliated with the genus *Marinobacter*.

The pure colonies were enriched in modified medium with 1.0 g/L of yeast extract and then collected for draft genome sequencing. Genomic DNA was extracted using the sucrose-Tris-EDTA (STE) method, and libraries were amplified with Agencourt SPRIselect kit (Beckman Coulter, USA) before quantification by quantitative PCR (qPCR) and sequencing on an Illumina NovaSeq 6000 platform (PE150). Raw reads were quality-filtered by Trimmomatic software (v0.40) (8) to remove low-quality sequences and adapter contamination. Draft assemblies were benchmarked with several tools; the final assembly was obtained with SPAdes v4.1.0 (9) (K-mers 99/127), which yielded the fewest contigs and highest N50 among tested settings. The assembly was polished with GapCloser (10) v1.12 to reduce gaps. Default parameters were used for all software unless otherwise specified.

The total raw reads of strain DUT-3 were 12,287,034 with a GC content of 57.91%. The assembly resulted in 24 contigs with approximately 110× coverage. The contig N50 length is 463,691 bp. The predicted genome length of strain DUT-3 was 3,826,671 bp. The Prokaryotic Genome Annotation Pipeline (PGAP) (11) was primarily used for genome annotation, with the self-constructed manganese-oxidizing gene database and CARD database (12) employed specifically for annotating manganese-oxidizing genes and antibiotic resistance genes (ARGs), respectively. Genome annotation predicted 3,817 protein-coding genes, containing 44 transfer RNAs, five rRNAs (3 5S, 1 16S, and 1 23S), and two small RNAs. The genome sequence was uploaded to TYGS server (https://tygs.dsmz.de/) for taxonomic classification and digital DNA-DNA hybridization (dDDH) calculation (13). Strain DUT-3 was classified as *Marinobacter* genus with a dDDH value (d4) of 29.3% between strain DUT-3 and *M. lipolyticus* SM19 (GenBank no. GCA_000397065.2)*,* which suggests strain DUT-3 is a potential novel species. The genome functional annotation further revealed two manganese oxidation genes (*stKatG* and *copA*) and several ARGs, for example, *sul3*, *tetT*, and *oqxA*, involved in sulfonamide, tetracycline, and fluoroquinolone antibiotic resistance. These genes in *Marinobacter* sp. DUT-3 suggest its ability of manganese oxidation and antibiotic resistance. Therefore, this study enhances our understanding of the mechanisms of manganese oxidation and antibiotic resistance in marine bacteria.

## Data Availability

The 16S rRNA sequence and draft genome sequence of *Marinobacter* sp. DUT-3 have been deposited to GenBank under accession numbers PV384102 and JBNRMG000000000, respectively. The raw sequence data have been submitted to the SRA database under accession number SRR32868370 (BioProject PRJNA1242082, BioSample SAMN47579270).
